# Photobiomodulation of Gingival Cells Challenged with Viable Oral Microbes

**DOI:** 10.1177/00220345241246529

**Published:** 2024-05-03

**Authors:** J. Tanum, H.E. Kim, S.M. Lee, A. Kim, J. Korostoff, G. Hwang

**Affiliations:** 1 Department of Preventive and Restorative Sciences, School of Dental Medicine, University of Pennsylvania, Philadelphia, PA, USA; 2 Department of Endodontics, School of Dental Medicine, University of Pennsylvania, Philadelphia, PA, USA; 3 Department of Medical Engineering, College of Engineering and Morsani College of Medicine, University of South Florida, Tampa, FL, USA; 4 Department of Periodontics, School of Dental Medicine, University of Pennsylvania, Philadelphia, PA, USA; 5 Center for Innovation & Precision Dentistry, School of Dental Medicine, School of Engineering and Applied Sciences, University of Pennsylvania, Philadelphia, PA, USA; 6 Chemical and Biomolecular Engineering College of Engineering, Yonsei University, Seoul, Republic of Korea

**Keywords:** antimicrobial resistance, host pathogen interactions, periodontal disease(s)/periodontitis, keratinocyte(s), biophotonics, reactive oxygen species

## Abstract

The oral cavity, a unique ecosystem harboring diverse microorganisms, maintains health through a balanced microflora. Disruption may lead to disease, emphasizing the protective role of gingival epithelial cells (GECs) in preventing harm from pathogenic oral microbes. Shifting GECs’ response from proinflammatory to antimicrobial could be a novel strategy for periodontitis. Photobiomodulation therapy (PBMT), a nonpharmacologic host modulatory approach, is considered an alternative to drugs. While the host cell response induced by a single type of pathogen-associated molecular patterns (PAMPs) was widely studied, this model does not address the cellular response to intact microbes that exhibit multiple PAMPs that might modulate the response. Inspired by this, we developed an in vitro model that simulates direct interactions between host cells and intact pathogens and evaluated the effect of PBMT on the response of human gingival keratinocytes (HGKs) to challenge viable oral microbes at both the cellular and molecular levels. Our data demonstrated that LED pretreatment on microbially challenged HGKs with specific continuous wavelengths (red: 615 nm; near-infrared: 880 nm) induced the production of various antimicrobial peptides, enhanced cell viability and proliferation, promoted reactive oxygen species scavenging, and down-modulated proinflammatory activity. The data also suggest a potential explanation regarding the superior efficacy of near-infrared light treatment compared with red light in enhancing antimicrobial activity and reducing cellular inflammation of HGKs. Taken together, the findings suggest that PBMT enhances the overall barrier function of gingival epithelium while minimizing inflammation-mediated breakdown of the underlying structures.

## Introduction

The oral cavity harbors a complex and diverse multitude of microorganisms. Commensals indirectly and directly prevent infections by supplying the host with essential nutrients, enhancing host defense mechanisms, and/or suppressing the emergence of pathobionts in oral biofilms, limiting their conversion to virulent biofilms (Negrini et al. 2021). The transition from health to disease involves the increased presence of “keystone pathogens” that, although at relatively low abundance, are capable of evading the host response and creating an environment that favors the growth of inflammophilic organisms, a process known as dysbiosis (Hajishengallis 2014). These organisms subsequently induce an exuberant inflammatory response that mediates the breakdown of the tooth-supporting structures and manifests itself as periodontitis. Such alteration of the commensal steady-state microflora and disruption of the equilibrium between the microbes and host response are key events in the pathogenesis of periodontitis (Costalonga and Herzberg 2014; Hajishengallis 2014; Hajishengallis et al. 2020).

Gingival epithelial cells (GECs) play a significant role in protecting the underlying structures that support teeth from the deleterious effects of oral microbes (Andrian et al. 2006). In addition, GECs serve as sentinels for the presence of pathogenic microbes (Lee and Yilmaz 2021). This is mediated through the interaction between microbial molecules known as pathogen-associated molecular patterns (PAMPs) with pattern-recognition receptors on host cells (Amarante-Mendes et al. 2018). For GECs, recognition of PAMPs on pathogens leads to the release of proinflammatory and antimicrobial molecules (Jain and Darveau 2010). In addition, it results in an enhanced expression of adhesion molecules on the surfaces of GECs, facilitating the directed migration of inflammatory cells toward the detected pathogens (Jain and Darveau 2010). Collectively, these events represent the initiation of the host’s innate gingival immune response to prevent microbial colonization of the underlying tissues. Modulation of the response of these cells to microbial challenge from one that is predominantly proinflammatory to one that is primarily antimicrobial could represent a novel approach for preventing or treating periodontitis.

In recent years, the administration of certain drugs (e.g., matrix metalloproteinase inhibitors and nonsteroidal anti-inflammatory agents) that can down-modulate distinct components of the host immune response has been proposed as an adjunct for treating periodontal disease (Preshaw 2018; Ren et al. 2023). Such “host modulatory therapy” can enhance the outcomes of conventional periodontal therapy by inhibiting the host response induced by bacterial pathogens. A potential alternative to the use of drugs is photobiomodulation therapy (PBMT), which involves exposing diseased or damaged tissues to low-intensity light that triggers photochemical changes within cells (Karu 2013; Dompe et al. 2020). PBMT has been used to manage many medical conditions, demonstrating its effectiveness in offering pain relief, reducing inflammation, and facilitating wound healing (Dompe et al. 2020; Kim et al. 2020; De Oliveira et al. 2022). Thus, PBMT could have potential as a nonpharmacologic form of host modulatory therapy.

In this study, we used an in vitro model that simulates direct interactions between host cells and intact pathogens. We hypothesized that PBMT on human gingival keratinocytes (HGKs) will alter their response to microbial challenges from one that is dominated by the production of proinflammatory molecules to one that is antimicrobial. Our data demonstrated that PBMT ameliorates the proinflammatory response of HGKs challenged with pathogenic oral microbes while enhancing the ability of the cells to mount a direct antibacterial response. These alterations in the HGKs’ response to microbial challenges could enhance the overall barrier function of gingival epithelium while minimizing inflammation-mediated breakdown of the underlying structures.

## Materials and Methods

The detailed materials and methods are presented in the Appendix.

## Results

### Challenge with Oral Microbes Reduces the Viability and Enhances the Proinflammatory Activity of HGKs

**Figure 1.**
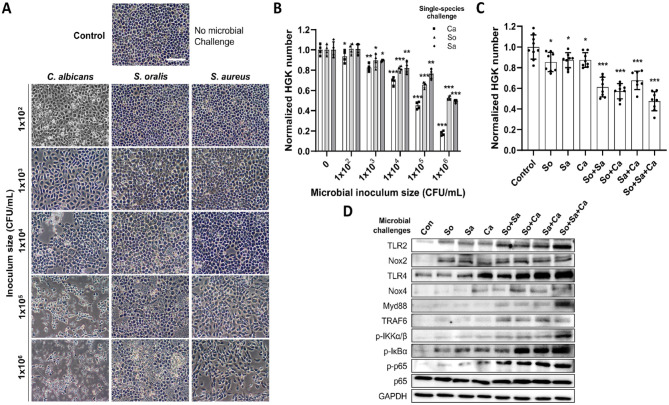
Effects of microbial challenges on human gingival keratinocytes (HGKs). (**A**) Optical microscopic images of human gingival keratinocytes under single-species microbial challenges depending on their populations. The scale bar represents 100 µm. (**B**) Normalized number of adhered HGKs under single-species microbial challenges, depending on the microbial colony-forming unit (CFU). (**C**) A normalized number of adhered HGKs under single-, double-, and triple-species microbial challenges. Inoculum size; *Streptococcus oralis* J22 and *Staphylococcus aureus* ATCC 6548 at ~1 × 10^3^ CFU/mL and *Candida albicans* 529L at ~1 × 10^2^ CFU/mL. (**D**) Western blotting analyses of TLR2, Nox2, TLR4, Nox4, MyD88, TRAF6, p-IKKα/β, p-IκBα, p-p65, and p65. GAPDH was used as a loading control. The *P* value was determined using a 2-tailed *t* test. *P* value (vs. Con): *P < 0.05, **P < 0.01, ***P < 0.001.

Before evaluating the impact of PBMT on the antimicrobial response of HGKs, we characterized the cellular response when co-cultured with increasing numbers of oral pathogens (Fig. 1A). The organisms used were those commonly detected in human periodontal lesions, *Streptococcus oralis* J22 (So) and *Candida albicans* 529L (Ca) (Cavalcanti et al. 2017; Hwang 2022), or *Staphylococcus aureus* ATCC6538 (Sa) from sites adjacent to dental implants with periimplantitis (Lafaurie et al. 2017). We chose those oxygen-tolerant microorganisms for co-culturing with HGKs. Microscopic analysis revealed a significant decrease in the number of adherent HGKs in response to co-culture with each microbe, with differing dose-response relationships (Fig. 1B). Particularly, Ca induced the largest loss of adherent cells over the entire range of added colony-forming units (CFUs), starting from ~1 × 10^2^ CFU/mL. A similar impact was observed from bacterial infections (So or Sa) at higher populations, starting from ~1 × 10^3^ CFU/mL. In addition to causing the most substantial loss of adherent cells, co-culture with a high number of CFUs of Ca also induced a dramatic change in the morphology of the remaining HGKs (Fig. 1A).

Subsequently, HGKs were exposed to various combinations of microbes to better mimic the situation in situ in which epithelial cells encounter multispecies biofilms. Using the lowest CFUs of each microbe that induced a significant decrease in the number of HGKs after 24 h of co-culture (i.e., ~1 × 10^2^ CFU/mL of Ca and ~1 × 10^3^ CFU/mL of So [or Sa]), mixed-species challenges caused a more significant reduction in adherent HGKs (Fig. 1C). Co-culture with all 3 microbes resulted in a cell loss equivalent to that observed with the highest individual bacterial populations (~1 × 10^6^ CFU/mL).

Western blotting was then performed to determine the expression of molecules involved in microbial recognition, induction of an HGK proinflammatory response, and participation in the effector phase of this response (Fig. 1D). Challenge with either bacterial species alone increased the synthesis of TLR2, and co-culture with Ca resulted in elevated levels of both TLR2 and TLR4. Combinations of multiple-species challenges induced enhanced production of TLR2 and TLR4. Interestingly, in the absence of Ca, the combination of So and Sa caused an increase in the level of TLR4 synthesis but to a lesser extent than observed in the presence of the fungus. All combinations of microbes led to enhanced levels of cytosolic proteins involved in TLR-mediated signaling, including Myd88, TRAF6, p−ΙΚΚα/β, p-ΙκBα, and p-p65.

Finally, all co-culture of HGKs with any of the microbes alone or in combinations resulted in increased levels of Nox2, a critical player in the generation of reactive oxygen species (ROS). Collectively, these findings indicate that the co-culture of viable oral microbes with HGKs primarily induces a proinflammatory response by the epithelial cells.

### PBMT Enhances the Viability and Inhibits the Proinflammatory Activity of Microbe-Challenged HGKs

**Figure 2.**
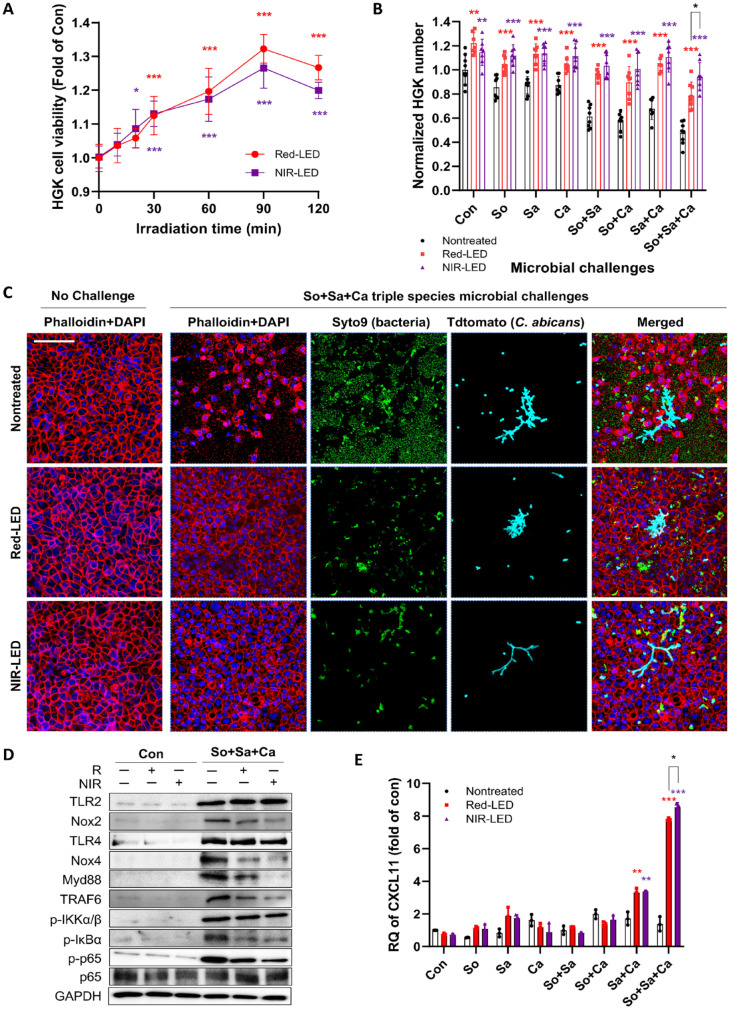
Effects of red or near-infrared pre-irradiation on human gingival keratinocytes (HGKs) against microbial challenges. (**A**) Effect of light irradiation (red [R]: 615 nm; near-infrared [NIR]: 880 nm) on the viability of HGKs without microbial challenges. (**B**) Normalized number of HGKs with or without light pre-irradiation (90 min) under various microbial challenges. Inoculum size; *S. oralis* J22 and *S. aureus* ATCC 6548 at ~1 × 10^3^ CFU/mL, and *C. albicans* 529L at ~1 × 10^2^ CFU/mL. Con: control group without light pre-irradiation. (**C**) Representative confocal images of phalloidin-red/DAPI-blue stained HGKs, exhibiting blue-fluorescent nuclei and red-fluorescent cytoskeleton. Syto 9 was used to stain bacteria, and Tdtomato was used to label *C. albicans*. The scale bar indicates 100 µm. (**D**) Western blotting analyses of expression levels of inflammatory-related proteins under triple-species challenges with or without light pre-irradiation. GAPDH was used as a loading control. (**E**) Expression levels of mRNA of CXCL11 after 24 h of single- or multiple-species microbial challenges in the presence/absence of photobiomodulation therapy. *P < 0.05, **P < 0.01, ***P < 0.001.

To investigate the effects of pretreatment with red (R-LED, 615 nm) and near-infrared (NIR-LED, 880 nm) light on HGKs, we initially determined how the light pretreatment affects the epithelial cells. The data revealed a significant increase in cell number (>130% vs. control; Fig. 2A) in response to PBMT, with the 90-min treatment duration showing the maximum efficacy. While 120-min exposure reduced efficacy (vs. 90 min), it remained higher than 60-min exposures. In addition, R-LED pretreatment promoted slightly higher HGK growth (vs. NIR-LED) at the same durations (Fig. 2A). Consequently, we chose the 90-min duration for further investigation into the efficacy of PBMT on HGK viability against microbial challenges. While 24 h exposure of HGKs to single or combinations of the microbes without PBMT decreased cell numbers by up to 50% (vs. control), pretreated HGKs numbers, in all cases, exhibited significantly higher cell numbers than nontreated HGKs (Fig. 2B). Notably, both R-LED and NIR-LED pretreatment maintained viable HGKs number similar to control HGKs (no PBMT, no microbial challenges), even under triple-species challenges. Intriguingly, NIR-LED pretreatment exhibited better efficacy in most microbial challenges.

HGKs co-cultured with or without a triple-species challenge were also visualized using a confocal microscope (Fig. 2C). In the absence of microbes, confluent monolayers with marked cell-cell contacts were observed. Conversely, under triple-species challenges, the tissue surface was predominantly covered by bacteria and fungus, disrupting HGK confluence and cell-cell contacts. Excitingly, red-LED or NIR-LED pretreatment almost completely retained HGK confluence and cell-cell contact features, with significantly lower colonized bacteria and fungus, particularly with NIR-LED pretreatment.

The effect of PBMT on the proinflammatory behavior of microbe-challenged HGKs was then evaluated using Western blotting. Co-culture with individual microbes elicited enhanced expression of TLR2, TLR4, and other proteins involved in TLR signaling (Fig. 2D). However, PBMT significantly reduced the expression levels of Myd88 and TRAF6 without affecting TLR2 and TLR4 levels. PBMT also markedly reduced Nox2 expression. Importantly, NIR-LED outperformed red-LED pretreatment in reducing those inflammatory cytokines. Quantified protein levels of TLR2, Nox2, TLR4, and Nox4 in the presence/absence of microbes and PBMT are shown in Appendix Figure S1.

Finally, the mechanisms through which PBMT down-modulates proinflammatory behavior were investigated by evaluating the expression of CXCLs. Co-culture of pretreated HGKs with the triple-species challenges significantly increased CXCL11 expression (Fig. 2E). Time-dependent experiments exhibited a stagnant expression up to 8 h, followed by an exponential increase after 12 h (Appendix Fig. S2). While increased expression levels of other CXCLs (i.e., CXCL9, 10, and 20) were observed, particularly for triple-species challenges, these were not as obvious as CXCL11 (Appendix Fig. S3).

Altogether, these results suggest that PBMT enhances HGKs’ ability to resist the cytotoxic effects of co-culture with oral microbes while simultaneously down-modulating the proinflammatory behavior of the epithelial cells.

### PBMT Enhances the Antimicrobial Behavior of HGKs Co-cultured with Viable Oral Microbes

**Figure 3.**
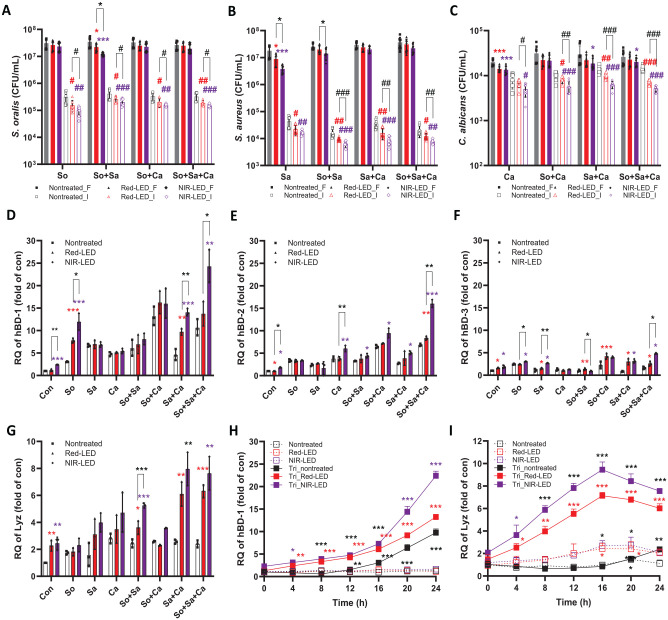
Changes in colony-forming units (CFU) and expression levels of antimicrobial peptides in human gingival keratinocytes (HGKs) in the presence and/or absence of photobiomodulation therapy (PBMT) and microbial challenges. (**A**–**C**) Microbial populations (CFU/mL) in the planktonic phase and on the HGKs after 24 h of incubation. F, free-floating supernatant; I, interface at HGKs. The *P* value was determined using a 2-tailed *t* test. *,^#^*P* < 0.05, **,^##^*P* < 0.01, ***,^###^*P* < 0.001. Expression levels of mRNA of (**D**) hBD-1, (**E**) hBD-2, (**F**) hBD-3, and (**G**) Lyz after 24 h of microbial challenges in the presence/absence of PBMT; HGKs were pre-irradiated for 90 min ahead of microbial challenges. The *P* value was determined using a 2-tailed *t* test. *P < 0.05, **P < 0.01, ***P < 0.001. Changes in expression levels of mRNA of (**H**) hBD-1 and (**I**) Lyz in each condition in a time-dependent manner. The *P* value was determined using a 2-tailed *t* test. *P < 0.05, **P < 0.01, ***P < 0.001 (vs. 0 h).

Next, we further investigated the antimicrobial response exhibited by HGKs due to PBMT and its impact on the viability of co-cultured microbes. Initially, we determined the number of adherent and planktonic microbial cells after 24 h of co-culture. Under all microbial challenge conditions, significantly fewer microbial cells were recovered from Red-LED or NIR-LED pretreated co-cultures (vs. nontreated cultures; Fig. 3A–C). The reduction of microbes adhered to HGKs was more pronounced than those found in the planktonic state. Notably, direct LED exposures (either red-LED or NIR-LED) did not affect the viability of microbial cells (Appendix Fig. S4), suggesting that PBMT enhanced the antimicrobial activity of HGKs.

Mucosal epithelial cells, including HGKs, participate in innate antimicrobial immunity via the secretion of antimicrobial peptides (AMPs) such as human β-defensins (hBD) and lysozyme (Lyz) (Mathews et al. 1999; Lokken-Toyli et al. 2021). To investigate whether PBMT enhances the expression of these AMPs, allowing cells to resist the effects of co-culture with the oral pathogens, we conducted quantitative reverse transcription polymerase chain reaction to quantify the expression levels of hBDs and Lyz (Fig. 3D–G) using relevant primers (Appendix Table S1). In the absence of microbial stimulation without LED pretreatment, there was extremely low-level expression of the evaluated genes. Co-culture of nontreated HGKs resulted in at least a 3-fold increase in the expression levels of hBD-1 and -2, whereas genes encoding hBD-3 and Lyz increased less than 3-fold (vs. control; Fig. 3D–G). Pretreated HGKs with red-LED or NIR-LED exhibited further increases in all genes except for hBD-3 (vs. control; Fig. 3D–G). The most dramatic increases were observed in pretreated HGKs co-cultured with triple-species challenge (up to 25-, 16-, and 8-fold for hBD-1, -2, and Lyz, respectively). In general, NIR-LED pretreatment induced larger increases in gene expression (vs. red-LED). Time-dependent analyses for hBD-1 revealed increasing expressions over time, while the expressions of Lyz peaked at 16 h of co-culturing and then decreased (Fig. 3H, I). Western blotting analyses of protein expression levels of hBD-1 and Lyz corroborated these trends (Appendix Fig. S5). These findings indicate that PBMT results in enhanced productions of hBD-1, -2, and Lyz by HGKs co-cultured with oral microbes, contributing to the enhanced survival of the epithelial cells under these conditions.

### PBMT Induces a Reduction in Intracellular ROS Levels and Enhanced Expression of Antioxidant-Related Genes in Microbe-Challenged HGKs

**Figure 4.**
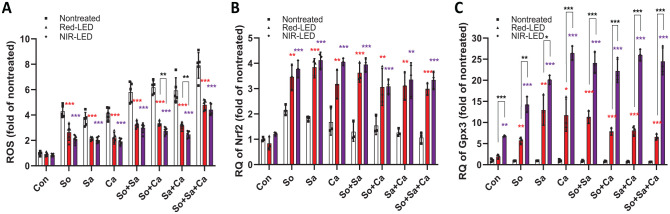
Effect of photobiomodulation therapy on intracellular levels of reactive oxygen species (ROS) and antioxidant-related gene expression. (**A**) Fold changes of the amount of intracellular ROS produced from human gingival keratinocytes with microbial challenges for 24 h. Fold changes of mRNA expression levels for (**B**) Nrf2 and (**C**) GPx3 genes. The *P* value was determined using a 2-tailed *t* test. *P < 0.05, **P < 0.01, ***P < 0.001.

Microbial challenge to HGKs led to an enhanced expression of Nox2, contributing to an increase in intracellular ROS levels associated with oxidative stress and eventual cell death. To investigate whether PBMT enhances HGK viability following exposure to oral microbes by inhibiting the accumulation of intracellular ROS, we conducted an analysis using DCF-DA. All types of microbial challenges induced significant increases in intracellular ROS levels (Fig. 4A). Single-species challenges increased the ROS level ~4-fold (vs. control with no PBMT). This increase was further enhanced to ~6- and ~8-fold under double- and triple-species challenges, respectively. In contrast, ROS levels in all conditions were significantly reduced when HKGs were pretreated with red-LED or NIR-LED (vs. nontreated control). Notably, NIR-LED pretreatment appeared to further reduce intracellular ROS production than red-LED did.

To better understand the ROS reduction mechanism by PBMT, we assessed the expression levels of antioxidant-related genes. Most of the antioxidant-related genes showed up-regulation in the presence of PBMT under all microbial challenge conditions (Appendix Fig. S6). Among them, the expression levels of Nrf2 (Fig. 4B) and Gpx3 (Fig. 4C) were significantly increased. Particularly, the level of Gpx3 was significantly up-regulated by NIR-LED pretreatment (vs. red-LED; Fig. 4C). These findings indicate that PBMT effectively reduces intracellular ROS in microbe-challenged HGKs by increasing ROS scavenging activity.

### PBMT Enhances Barrier Function of Microbe-Challenge HGKs

**Figure 5.**
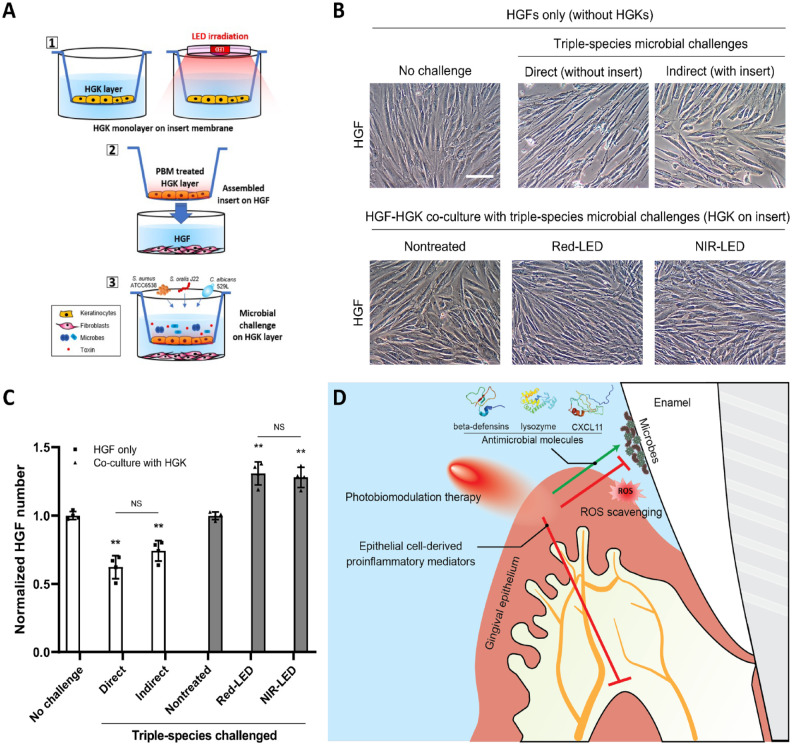
Interaction between human gingival keratinocytes (HGKs) and human gingival fibroblasts (HGFs) in the absence/presence of polymicrobial challenges and photobiomodulation therapy. (**A**) A schematic diagram describing the co-culture system using an insert transmembrane. (**B**) Images of HGFs with (bottom lane) or without (upper lane) HGKs. No challenge, HGFs without microbial challenges; direct, HGFs directly exposed to microbial suspension without insert transmembrane; indirect, HGFs indirectly exposed to microbial suspension only on insert transmembrane; nontreated, HGFs (below insert transmembrane) with nontreated HGKs exposed to microbial suspension (on insert transmembrane); red-LED, HGFs (below insert transmembrane) with pretreated HGKs by red-LED exposed to microbial challenges (on insert transmembrane); NIR-LED, HGFs (below insert transmembrane) with pretreated HGKs by NIR-LED exposed to microbial challenges (on insert transmembrane). The scale bar represents 100 µm. (**C**) The number of HGFs in each condition. NS: not significant, **P < 0.01. (**D**) A schematic diagram describing the role of photobiomodulation therapy on the interactions between bacteria and gingival epithelium cells.

To investigate the effectiveness of pretreated HGKs in enhancing the barrier function of keratinocytes to protect underlying human gingival fibroblasts (HGFs) against noxious external stimuli, we constructed a 3-dimensional co-culture system using transmembrane inserts (Fig. 5A). Either direct contact of HGFs with triple-species challenge (without a transmembrane insert) or indirect contact (microbial cells existed only in the transmembrane phase) induced morphological changes in HGFs, making them bipolar and elongated (Fig. 5B). In addition, there was a decrease in the number of microbe-challenged HGFs, particularly under conditions of direct contact with microbial cells (Fig. 5C). When HGKs and HGFs were cultured, both red-LED and NIR-LED pretreatment of HGKs significantly improved the ability of the microbe-challenged epithelial cells to protect the underlying HGFs from the deleterious effects of the microbes (Fig. 5B, C).

## Discussion

PBMT has emerged as a potential alternative to chemical agents (including antibiotics) for treating diseases with microbial and/or inflammatory origins (de Freitas and Hamblin 2016). With regard to periodontitis, studies have shown that GECs exposed to PBMT, when challenged with lipopolysaccharide (LPS) or heat-killed periodontal pathogens, enhance their viability and proliferation rate and alter the production of proinflammatory molecules (Pansani et al. 2018). While the host cell response induced by a single type of PAMP has been widely studied (Maldonado et al. 2016; Dickson and Lehmann 2019), this model does not address the cellular response to intact microbes, presenting multiple PAMPs and other molecules that might modulate the response. Furthermore, the response of epithelial cells to PBMT could vary depending on the types of stimulants (e.g., microbes and their metabolic products). This study aimed to evaluate the effect of PBMT on HGKs in response to challenges with viable oral microbes at the cellular/molecular levels to better understand the mechanisms through which the technology might enhance the overall barrier function of gingival epithelium while minimizing inflammation-mediated breakdown of the underlying structures.

Direct contact between HGKs and the microorganisms led to elevated ROS generation and the induction of a proinflammatory response via the TLR/Nox/NFκB pathway (Fig. 1D). Particularly, mixed-species infection increased the expression of TLR2/4, Nox2/4, and Toll/IL-1R domain-containing adaptor proteins such as MyD88 and TRAF6. This observation is consistent with previous studies demonstrating that TLR2/4 recruits MyD88 upon contact with microbial products (LPS or heat-killed bacteria), linking it to IL-1R–associated kinase via TRAF6, which triggers the activation of NFκB and the production of proinflammatory cytokines and chemokines that can dictate the outcome of innate immune responses (Kawai and Akira 2010; Sameer and Nissar 2021). Our data demonstrated that the HGK response is not solely dependent on LPS exposure, as we observed the proinflammatory response with gram-positive bacteria and a fungal organism.

The mitochondrial cytochrome c oxidase is capable of absorbing red/near-infrared lights that facilitate adenosine triphosphate production, thereby inducing transcription factors and modulating ROS production (Sun et al. 2018). As a consequence, photobiomodulation increases cell proliferation/migration; modulates the levels of cytokines, growth factors, and inflammatory mediators; and increases the amount of oxygen in the tissues. Similarly, we found that the number of HGKs in pretreated cultures was significantly higher (vs. nontreated cultures; Fig. 2A). Furthermore, PBMT moderated the host inflammatory response to microbial challenges by down-regulating Nox2/4, MyD88, and TRAF6, which resulted in a decrease in NFκB levels. In addition, CXCL11 is known to recruit CD4-positive T cells to sites of inflammation and under certain conditions will induce their differentiation into regulatory T cells that can down-modulate the inflammatory reaction (Tregs) (Zohar et al. 2018). A marked reduction in Nox2 and a dramatic increase in CXCL11 expression by pretreated HGKs represent potential explanations for the inhibition of their proinflammatory response to microbial challenge. Notably, CXCL11 has been shown to exhibit antibacterial activity (Cole et al. 2001). Thus, it is feasible that in addition to recruiting and inducing the differentiation of Tregs, the antimicrobial activity of CXCL11 might further protect the HGKs from the effects of the oral microbes. Further investigations are necessary to identify the mechanism underlying this observation.

Beyond cell proliferation and the proinflammatory response, PBMT directly mediated antimicrobial immunity via the production of AMPs and extracellular ROS. Among many, hBDs are expressed in response to bacterial stimuli or inflammation (Chung and Dale 2004), exhibiting antimicrobial effects (Dale et al. 2001). In addition, lysozyme (Lyz), one of the host defense peptides, can kill bacteria through the catalytic hydrolysis of cells (Gill et al. 2011). Interestingly, Lyz can operate synergistically with hBD-2 and hBD-3 (Lippross et al. 2012); Lyz can crack the bacteria cell wall polysaccharide chain, enhancing the entry of hBD into the cell (Travis et al. 2001). In this study, we observed significant increases in hBD-1, hBD-2, and Lyz expression levels (Fig. 3), particularly under triple-species challenges. Also, PBMT effectively reduced intracellular ROS in microbe-challenged HGKs by increasing ROS scavenging activity. This mechanism could be involved in the down-modulation of the proinflammatory activity of microbe-challenged HGKs by interfering with ROS-mediated activation of the NFκB signaling pathway and assembly of the NLRP3 inflammasome (Liu et al. 2017; Palazon-Riquelme and Lopez-Castejon 2018). These findings indicate that PBMT of HGKs prior to culture with oral microbes causes an alteration in the response of the epithelial cells from one that is predominantly proinflammatory to the antimicrobial response.

Interestingly, red-LED pretreatment was more effective than NIR-LED in stimulating HGK proliferation, whereas NIR-LED appeared to more effectively activate the cell’s antimicrobial activity (Fig. 2). The antioxidant enzyme Gpx, crucial for detoxifying ROS and shielding epithelial cells from oxidative damage (Ottaviano et al. 2009), and the transcription factor Nrf2, involved in the antioxidant response (Huang et al. 2015), were up-regulated by PBMT. In particular, Gpx3 exhibited a remarkable up-regulation under NIR-LED pretreatment (vs. red-LED; Fig. 4), potentially explaining the higher antimicrobial activity and reduction of cellular inflammation in HGKs with NIR-LED.

Finally, keratinocytes are known to act as a protective barrier against microbial invasion of underlying tissues as well as to activate fibroblasts to produce a variety of soluble mediators (Shephard et al. 2004; Ren et al. 2019). Thus, a 3-dimensional co-culture system was developed to assess the capacity of HGKs to protect underlying HGFs from microbial challenge. In the absence of HGKs, HGFs were severely damaged by either direct contact with microbial cells or indirectly by their products (Fig. 5). In contrast, the viability of HGFs significantly improved even under triple-species challenges when co-cultured with HGKs. The numbers and confluence of HGFs were dramatically increased when the HGKs were pretreated with LEDs. The findings suggest that PBMT enhances the overall barrier function of gingival epithelium while minimizing inflammation-mediated breakdown of the underlying structures.

In summary, we comprehensively evaluated the effect of PBMT on the response of HGKs to viable oral microbes challenge at the cellular/molecular levels. The data demonstrate that light pretreatment of microbially challenged HGKs induced the production of various AMPs in addition to well-known PBMT effects on cell viability/proliferation, ROS scavenging, and down-modulation of proinflammatory activity (Fig. 5D). Our data also suggest a potential explanation regarding the superior efficacy of NIR-LED compared with red-LED in enhancing antimicrobial activity and reducing cellular inflammation of HGKs. While the findings of this study exhibit the potent benefit of using phototherapy, further studies are warranted to enhance its clinical efficacy. Since bacteria and fungi coexist in the oral cavity and reciprocally enhance one another’s pathogenic potential, for example, it will be useful if irradiation conditions can be identified that stimulate HGKs to produce both antibacterial and antifungal proteins such as histatins (Buda et al. 2020). In addition, it is certainly of interest to evaluate the efficacy of postchallenge or intermittent treatment during challenges. Furthermore, determining the time course of effect with an elongated experimental period using an organoid model would be necessary to better evaluate the efficacy. Finally, the cellular and molecular responses can be altered by varying the PBMT parameters (e.g., power [intensity], energy [density], spot area, operation mode) and/or the type of pathogens. Further understanding of the mechanism and the efficacy of PBMT addressing those perspectives may broaden the applicability for oral and extraoral microbially induced inflammatory conditions.

## Author Contributions

J. Tanum, contributed to data analysis and interpretation and drafted and critically revised the manuscript; H.E. Kim, contributed to data acquisition, analysis, and interpretation and drafted the manuscript; S.M. Lee, A. Kim, contributed to data acquisition and drafted the manuscript; J. Korostoff, contributed to data analysis and interpretation, critically revised the manuscript; G. Hwang, contributed to conception, design, data analysis, and interpretation and drafted and critically revised the manuscript. All authors gave final approval and agree to be accountable for all aspects of the work.

## Supplemental Material

sj-docx-1-jdr-10.1177_00220345241246529 – Supplemental material for Photobiomodulation of Gingival Cells Challenged with Viable Oral Microbes Supplemental material, sj-docx-1-jdr-10.1177_00220345241246529 for Photobiomodulation of Gingival Cells Challenged with Viable Oral Microbes by J. Tanum, H.E. Kim, S.M. Lee, A. Kim, J. Korostoff and G. Hwang in Journal of Dental Research
