# Supplementary material for: Photobiomodulation of Gingival Cells Challenged with Viable Oral Microbes
Source: J Dent Res. 2024 May 3;103(7):745–54. doi: 10.1177/00220345241246529 (PMC11191660; doi:10.1177/00220345241246529)
Supplement: sj-docx-1-jdr-10.1177_00220345241246529 – Supplemental material for Photobiomodulation of Gingival Cells Challenged with Viable Oral Microbes [file sj-docx-1-jdr-10.1177_00220345241246529.docx]

**Appendix for Photobiomodulation of gingival cells challenged with viable oral microbes**

Junjira Tanum^1,†^, Hye-Eun Kim^1,†^ Su-Min Lee^2^, Albert Kim^3^, Jonathan Korostoff^4,*^, Geelsu Hwang^1,5,6,*^

^1^Department of Preventive and Restorative Sciences, School of Dental Medicine, University of Pennsylvania, Philadelphia, PA 19104, USA

^2^Department of Endodontics, School of Dental Medicine, University of Pennsylvania, Philadelphia, PA, 19104 USA

^3^Department of Medical Engineering, College of Engineering and Morsani College of Medicine, University of South Florida, Tampa, FL, 33620 USA

^4^Department of Periodontics, School of Dental Medicine, University of Pennsylvania, Philadelphia, PA, 19104 USA

^5^Center for Innovation & Precision Dentistry, School of Dental Medicine, School of Engineering and Applied Sciences, University of Pennsylvania, Philadelphia, PA 19104, USA

^6^Chemical and Biomolecular Engineering College of Engineering, Yonsei University, Seoul, 03722, Republic of Korea

^†^ These authors contributed equally to this work.

Corresponding Authors:

*Geelsu Hwang, email: [geelsuh@upenn.edu](mailto:geelsuh@upenn.edu); Jonathan Korostoff, email: [jkorosto@upenn.edu](mailto:jkorosto@upenn.edu)

**Materials and Methods**

**Microorganisms and microbial culture conditions**

Candida albicans 529L, a clinical oral isolate (Cuomo, Fanning et al. 2019), and *Streptococcus oralis* J22 (Kim, Ito et al. 2022), *Staphylococcus aureus* ATCC6528 were used for the microbial challenge. Microbial stocks were stored at −80°C in tryptic soy broth containing 50% glycerol before use. All strains were grown to mid-exponential phase (optical densities at 600 nm of 0.8 (*C. albicans*) and 1.0 (*S. oralis* and *S. aureus*), respectively) in ultrafiltered (10 kDa molecular mass cutoff; Millipore, Billerica, MA, USA) yeast-tryptone extract broth containing 2.5% tryptone and 1.5% yeast extract (UFTYE; pH 5.5 and 7.0 for yeast and bacteria, respectively) with 1% (wt/vol) glucose at 37°C and 5% CO_2_.

**PBMT setting**

In our previous study (Kim, Islam et al. 2020), we set the emission angle and the light irradiance by the LED platform to be uniform across all wavelengths. To have uniform light coverage on entire cells, LEDs with an emission angle of 120° or larger were chosen. At the given angle and distance (the distance between the LED and the bottom of the cell plate is 2.3 mm), the spot diameter is 8 mm (or a spot area of 50 mm^-2^). We used a calibrated photodetector (FDS1010, Thorlabs) with a detection area of 1 cm^-2^, capable of measuring the light irradiance of our LED spot area. The light irradiance at the level of cells was set to be 0.8 mW cm^–2^, which induced minimal temperature increase (<1.1 °C; measured ≈3 weeks). The input driving voltage was regulated individually for each wavelength. The voltages to irradiate 0.8 mW cm^–2^ were 2V and 4.3V for near-infrared and red, respectively. The energy density that cells received was up to 6.00 J cm^–2^ when irradiated for 120 min. Furthermore, we calculated the wavelength photon fluency, which is a newly proposed metric for dosimetry (Young, Maximiano and Arany 2022) (see Appendix Table S2).

**Human gingival cell culture and optimization of PBMT**

Human gingival keratinocytes (HGKs) were kindly provided by the laboratory of Dana T. Graves at the University of Pennsylvania and cultured in KGM^TM^ Gold Keratinocyte Bullet Kit (Cat# 00195769, Lonza, Basel, Switzerland) at 37°C in a humid atmosphere of 5% CO_2_. Primary human gingival fibroblasts (HGFs) were collected from four periodontally and systemically healthy donors (no medication or general disease) having no signs of periodontal attachment loss and no bleeding on probing. All participants were patients of the University of Pennsylvania School of Dental Medicine. The study was reviewed and approved by the Institutional Review Board of the University of Pennsylvania (protocol #825886). Written informed consent was obtained from the participants. Obtained gingival tissues were cut into small 2 mm × 2 mm pieces and then incubated for 6 h at 37°C with 1 mg/mL Collagenase/Diapase (Collagenase: 0.1U/mL, Dispase: 0.8U/mL, 10269638001, Millipore Sigma, USA). Thereafter, the epithelium was collected by mechanical separation and treated with 0.2% trypsin for 10 min at 37°C, and then the cell suspension was centrifuged for 10 min at 120 g. After washing, the cells were seeded in collagen-coated plates and cultured in Fibroblast Basal Medium (ATCC PCS-201-030) supplemented with Fibroblast Growth Kit–Low Serum (ATCC PCS-201-041) at 37°C in a humidiﬁed atmosphere of 95% air and 5% CO_2_.

To test the efficacy of PBMT, HGKs were seeded at 5 × 10^4^ cells per well in a 24-well plate and grown for 24 h at 37 °C. After 24 h, the cells were washed with 1× PBS and replaced medium without growth factors. Then, the cells were placed on the 3D printed LED platforms to be exposed to red (λ = 615 nm) or near-infrared (λ = 880 nm) LED for various times (up to 120 min) as described in the previous reports (Kim, Islam et al. 2020, Park, Islam et al. 2020, Dhall, Tan et al. 2022). After the light treatment, the cells were incubated for an additional 24 hours. The next day, 10 μl of 3-(4,5-dimethyl-2-thiazolyl)-2,5-diphenyl-2H-tetrazolium bromide (MTT) reagent (Sigma-Aldrich, St. Louis, MO, USA) was added to 90 μL of fresh serum-free KBM-2 medium. Samples were left for 5 h. Well volumes were then replaced with dimethyl sulfoxide (DMSO; Sigma-Aldrich, St. Louis, MO, USA). The absorbance was measured at 490 mm using a microplate reader (SpectraMax M2, Molecular Device, USA). Percentage cell viability was calculated from the absorbance readings. Three independent experiments were conducted at least triplicate. Once a well-established HGK monolayer was confirmed on a 24-well tissue-culture plate, HGK culture media were refreshed KBM-2 medium without growth factors and antibiotics.

**Microbial challenge to HGK monolayers**

HGKs were seeded on the bottom of tissue-culture polystyrene wells at a cell density of 5 x 10^4^ cells per well (24 wells per plate) in 1 ml KBM-2 medium with 0.15 μM CaCl_2_ and grown for 24 h. The next day, the medium changed to fresh KBM-2 medium without CaCl_2_ and incubated for 48 h. Then, media were refreshed by 900 ml of the medium without antibiotics and 100 ml medium containing 10^2^, 10^3^, 10^4^, 10^5^, and 10^6^ of *S. oralis* J22, *S. aureus* ATCC6548, or *C. albicans* 529L per ml, added. Medium without bacteria was added as a control. After co-culturing for 24 h, cells were washed with phosphate-buffered saline (PBS, 10 mM potassium phosphate, 0.15 M NaCl, pH 7), and the attached HGK monolayer on plates was examined by an optical microscope (Nikon, Japan). The number of HGKs was quantitatively analyzed using Image J. The HGKs were fixed with 4% paraformaldehyde and stained with cytoskeleton stain (DyLight ™ 650, phalloidin, Thermo) and blue-fluorescent nucleus stain (DAPI, Thermo) for confocal microscopy. In a separate experiment, we examined the spatial organization of HGK-microbe interaction using confocal laser scanning microscopy (CLSM). Briefly, bacterial cells were stained with 2.5 μM SYTO 9 green fluorescent nucleic acid stain (Molecular Probes) and C. albicans cells were stained with concanavalin A (ConA) lectin conjugated with tetramethylrhodamine at 40 μg/mL (Molecular Probes) (Kim, Liu et al. 2020, Kim, Dhall et al. 2021). DAPI and phalloidin were used to stain HGKs. Confocal images were obtained using an upright single-photon confocal microscope (LSM800, Zeiss) with a ×20 (numerical aperture, 1.0) water objective. All experiments were conducted four times; each condition contained 4 biological samples.

**Microbial counting in Cell-free supernatant and Cell-invaded/attached Microorganism**

After PBMT on a well-established HGK monolayer, *S. oralis* J22 and *S. aureus* ATCC 6548 at 10^3^ CFU mL^-1^, and *C. albicans* 529L at 10^2^ CFU mL^-1^ are challenged as single, double, or triple species mixture. After 24 h, the cell-free supernatant (CFS) was harvested, the remaining microbe on HGKs or plates were gently washed three times with 1xPBS, and this washing solution was added to the CFS to make a final volume of 5 mL. This CFS was sonicated on ice (3 × 10 s) at 30 W (Vibra Cell model 375; Sonics and Materials, Danbury, CT, USA) to break down bacterial chains and microbial aggregates. Microorganisms in CFS were harvested by centrifugation (5000 × g, 10 min, 4 °C) and resuspended in 1 mL of 1x PBS. Microorganisms invaded or firmly attached to HGKs were harvested by trypsinization with HGKs, then sonicated to break down and harvest microbial-cell aggregates in the same condition as CFS. To count the number of microbial CFU, the resuspending solution was taken and the number of CFU present was counted after serial dilution and plating on Trypticase™ Soy Agar with 5% Sheep Blood (TSA II). Enumeration was done after incubation at 37 °C for 48 h and data was expressed as log CFU ml^-1^.

**Western Blotting**

Cells were lysed in cold piece RIPA buffer (Cat# 89900, Thermo Fisher Scientific, Rockford, IL) containing protease inhibitor cocktail (P8340, Sigma, St. Louis, MO). The whole-cell lysates (100 µg per lane) were separated by SDS-PAGE and detected with primary antibodies. Primary antibodies specific for TRAF6, NFkB, pNFkB, and GAPDH were purchased from Cell Signaling Technology (Beverly, MA). Antibodies for TLR2, NOX2, TLR4, and MyD88 were obtained from Santa Cruz Biotechnology (Santa Cruz, CA). Antibody for NOX4 was obtained from Novus (St. Charles, MO). The secondary antibodies used were anti-rabbit IgG and anti-mouse IgG from Santa Cruz Biotechnology. Bound secondary antibodies were detected using SuperSignal West Pico Chemiluminescent Substrate (Thermo Fisher Scientific). Equal loading was assessed using an anti-GAPDH antibody to normalize the amounts of total protein. The protein levels were determined by ImageJ software.

**Intracellular ROS assay**

After microbial challenge for 24 h, HGKs were rinsed with 1xPBS gently and subjected to the ROS assay. This assay was also performed on the various combinations of HGKs under microbial challenges in the absence/presence of Red-LED and NIR-LED pre-treatment. To measure the produced ROS, the HGKs were incubated with 1 µM of 2’,7’-dichlorodihydrofluorescein diacetate (H2DCF-DA; Sigma) for 45 min at 37 °C. After DCF-DA incubation, cells were washed with PBS, and DCF fluorescence intensity was monitored using a spectrofluorometer (SpectraMax M2, Molecular Device, USA) with excitation at 485 nm and emission at 530 nm, respectively.

**Quantitative Real-Time PCR**

After microbial challenges on pre-treated HGKs, the cells were harvested and total RNA was isolated with an RNeasy Mini kit (Qiagen, Valencia, CA) for qRT-PCR. To synthesize cDNA from total RNA, a High-Capacity cDNA Reverse Transcription Kit (Applied Biosystems, Foster City, CA) was used according to the manufacturer's protocol. Gene-specific RT-PCR primers were selected from the mRNA sequences obtained from NCBI's reference sequence database (http://www.ncbi.nlm.nih.gov/refseq/). The primer sets for quantitative real-time PCR in this study are described in Appendix Table S1. mRNA levels of anti-microbial peptides and ROS scavengers were quantified by real-time PCR using a PowerUp SYBR Green Master Mix (Applied Biosystems, Foster City, CA) and Bio-Rad CFX96 thermal cycler, following the manufacturer's protocol. Each sample was tested in triplicate, and human GAPDH was used as a control for the normalization of RNA expression.

**3D culture system**

To establish a 3D-tissue model, 1x10^4^ HGFs were seeded on the bottoms of 24-well plates and 1x10^4^ HGK cells were seeded on the top of a transwell (0.4 μm pore size, Costar, 3413, New York, NY, USA) and then cultured separately in the appropriate medium. After 72 h cultivation, HGKs were treated with PBM and assembled on HGFs. Then, microbes were challenged only into the upper insert with HGK monolayer for 24 h. Medium without microbes was added as a control. An insert without an HGK monolayer was added to observe whether microorganisms penetrated the transmembrane and invaded the HGFs. After 24 h microbial challenge, HGK and HGF cells were very gently washed with phosphate-buffered saline (PBS, 10 mM potassium phosphate, 0.15 M NaCl, pH 7), and the attached HGFs on plates were examined by an optical microscope (Nikon, Japan). The number of HGFs was quantitatively analyzed using Image J.


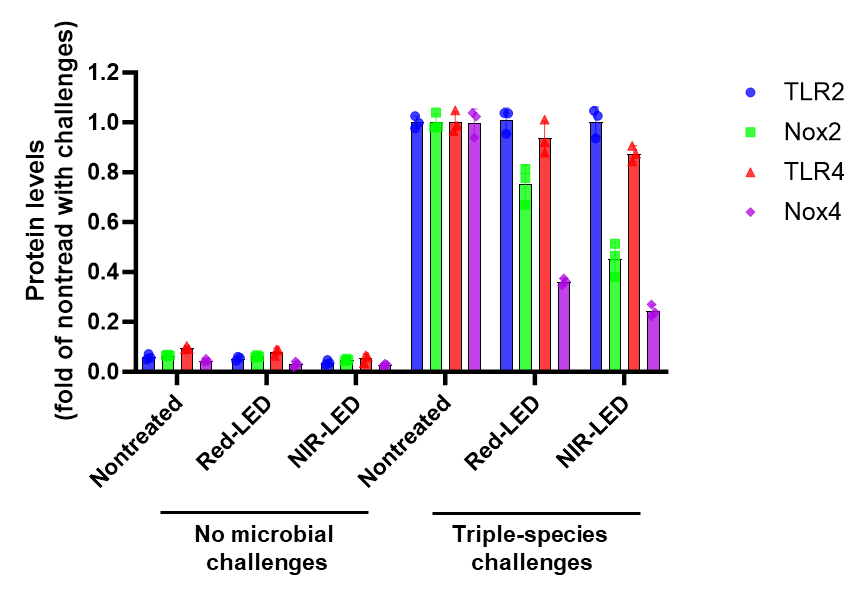


Appendix Figure S1. Quantified protein levels of TLR2, Nox2, TLR4, and Nox4 in the presence/absence of microbial challenges and PBMT.


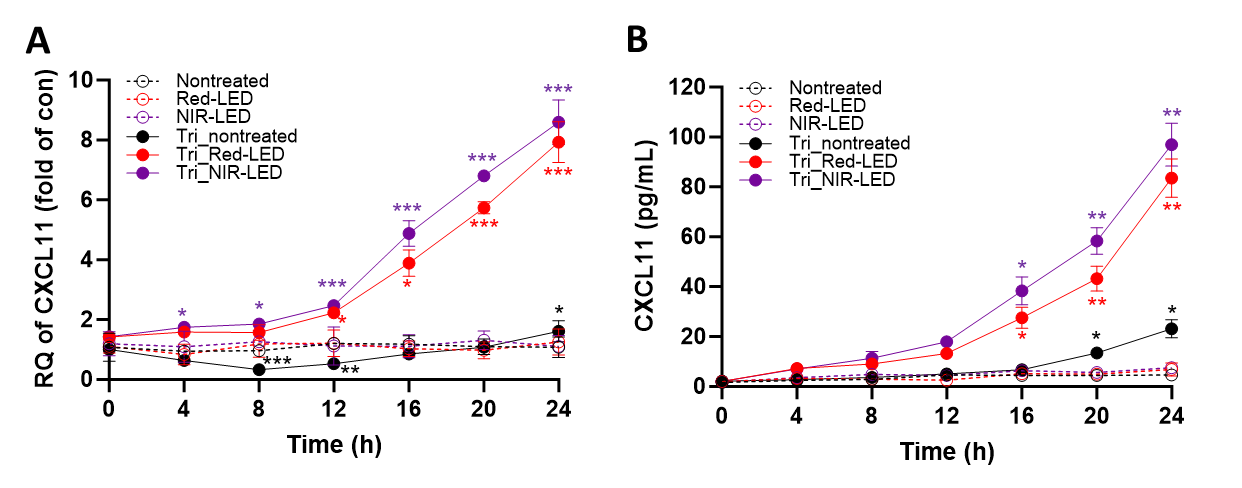


Appendix Figure S2. Changes in expression levels of mRNA of (A) CXCL11in a time-dependent manner. p-value was determined using a two-tailed t-test. *p<0.05, **p<0.01, ***p<0.001 (vs 0 hr). (B) Secreted concentrations of CXCL11 in each condition in a time-dependent manner measured by ELISA. p-value was determined using a two-tailed t-test. *p<0.05, **p<0.01 (vs 0 hr).


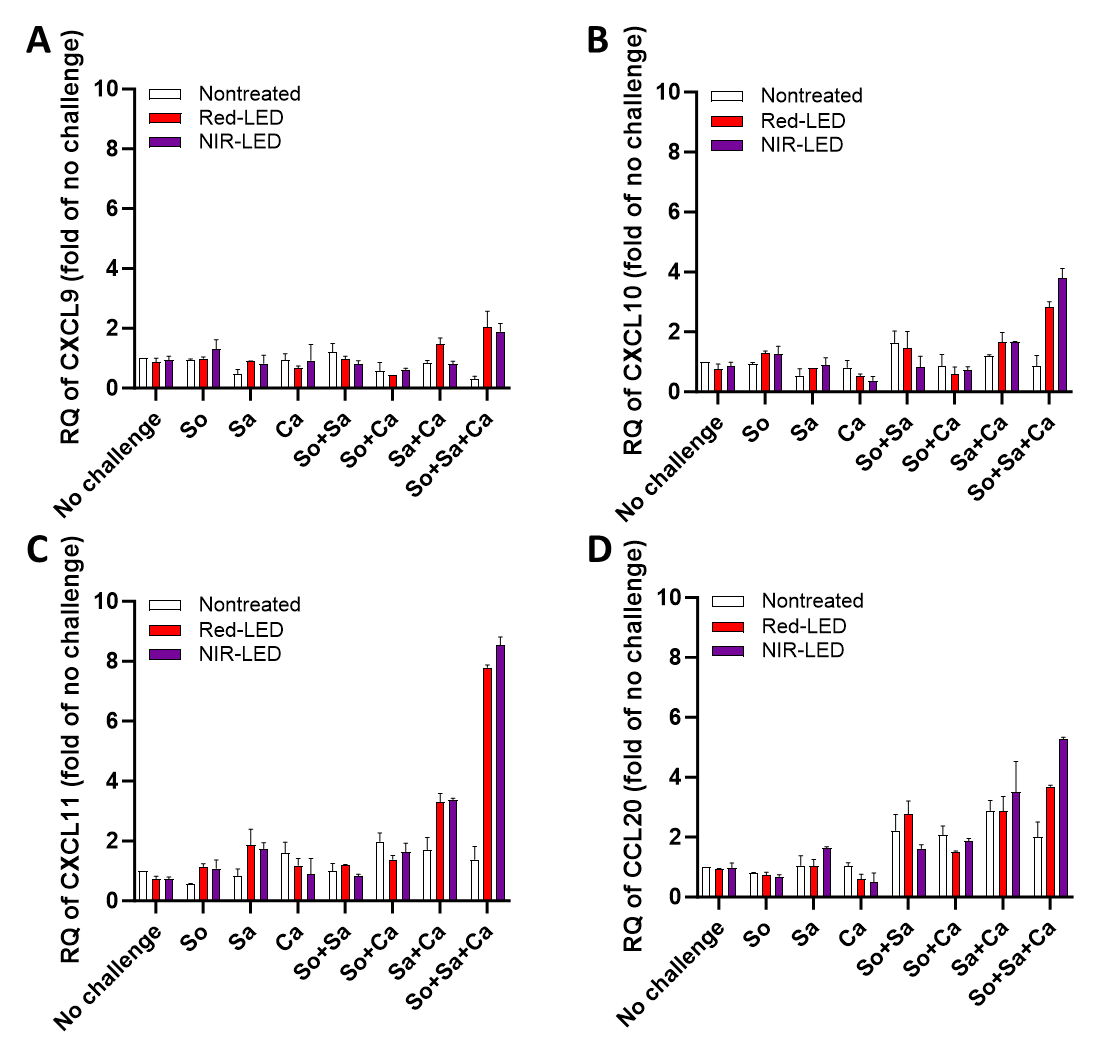


Appendix Figure S3. Expression levels of mRNA of (A) CXCL9, (B) CXCL10, (C) CXCL11, and (D) CCL20 after 24 h of single or multiple-species microbial challenges in the presence/absence of PBMT; White column: non-treated control, Red column: pre-treated with 625 nm red LED (Red-LED), Purple column: pre-treated with 880 nm near-infrared LED (NIR-LED), HGKs were pre-treated for 90 min ahead of microbial challenges. p-value was determined using a two-tailed t-test. *p<0.05, **p<0.01, ***p<0.001 (vs Con). #p<0.05, ##p<0.01, ###p<0.001 (Red vs NIR).

Appendix Figure S4. CFU of employed microbial cells in the absence/presence of PBMT.


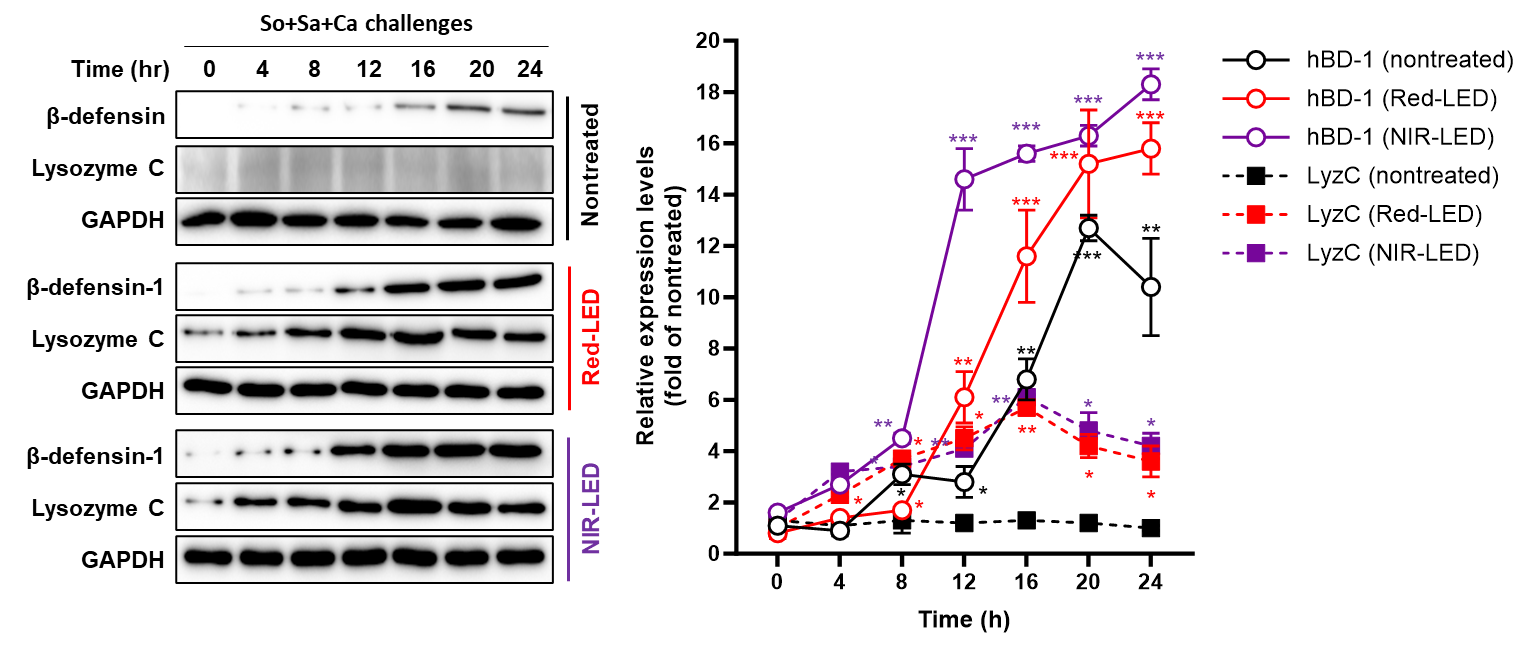


Appendix Figure S5. Western blotting analyses of proteins expression levels of hBD-1 and Lysozyme C in each condition in a time-dependent manner. GAPDH was used as a loading control. p-value was determined using a two-tailed t-test. *p<0.05, **p<0.01, ***p<0.001 (vs 0 hr)


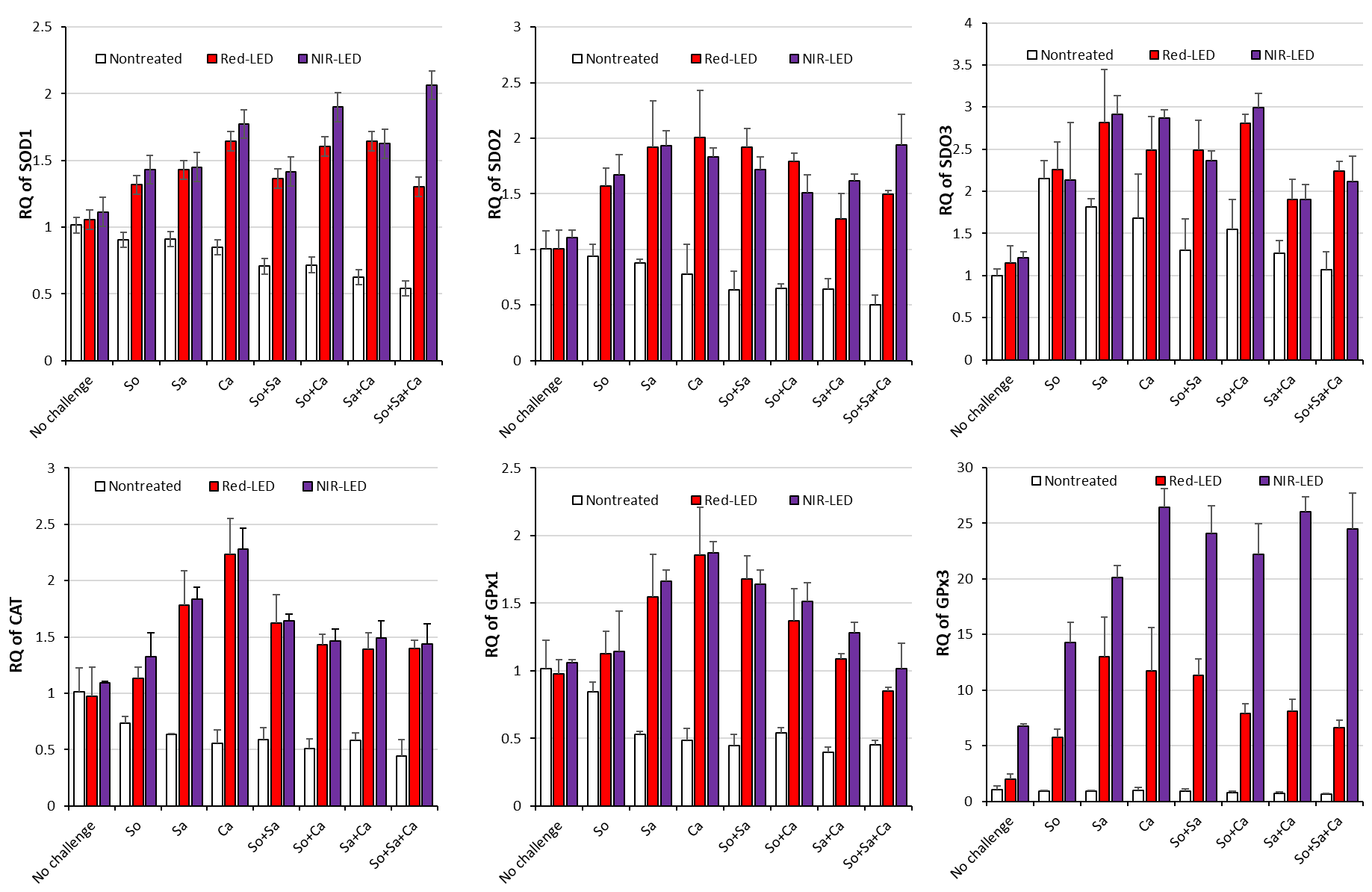


Appendix Figure S6. Changes in expression levels of antioxidant-related genes in each condition.

Appendix Table S1. List of primers for qRT-PCR in this study.


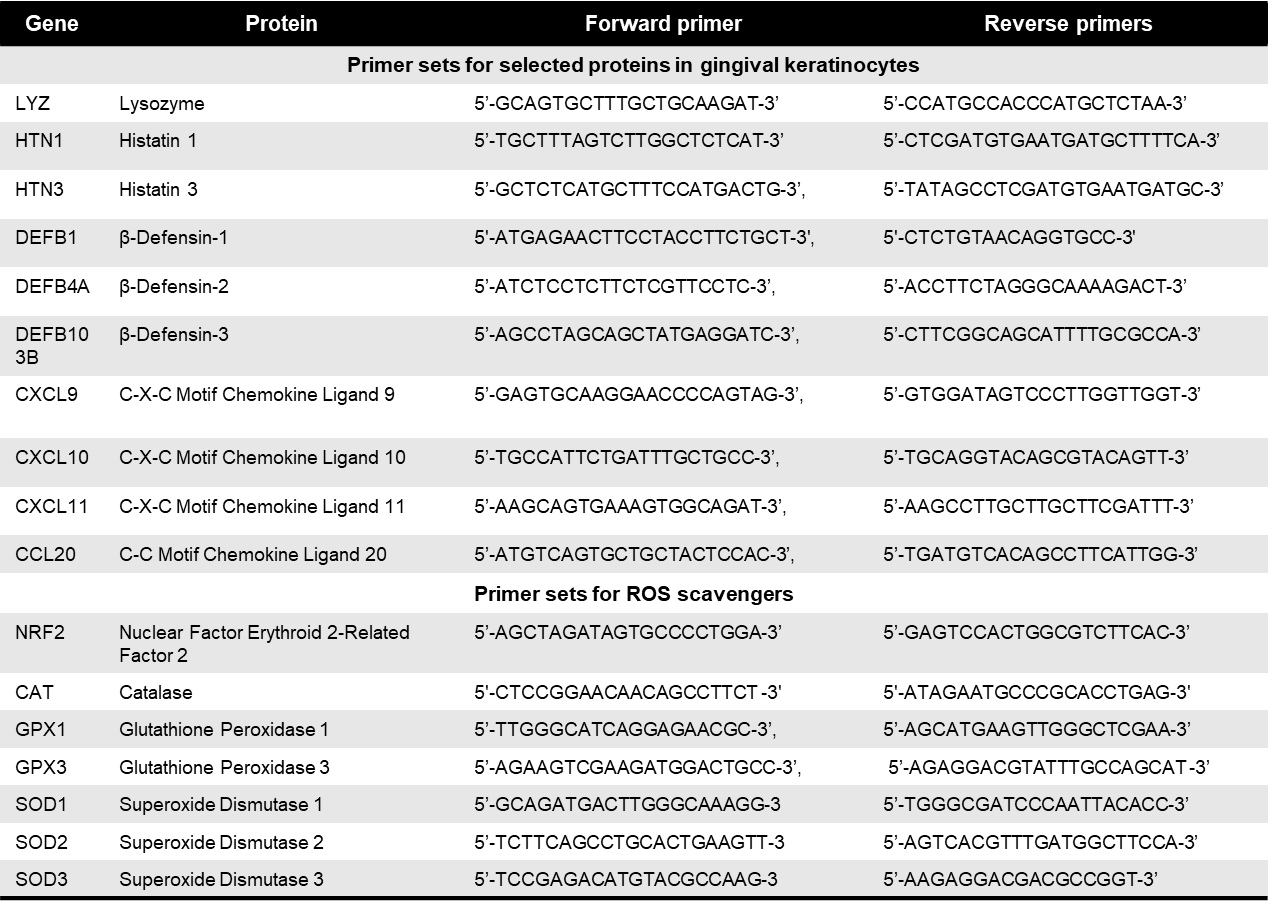


Appendix Table S2. Photobiomodulation dosimetry (wavelength photon fluency) used in this study.

| NIR-LED; wavelength 880 (nm) | | | | | |
| --- | --- | --- | --- | --- | --- |
| Time (min) | **Irradiance (mW/cm2)** | **Energy density (J/cm2)** | **eV** | **Photonic fluence (p.J/cm2)** | **Einstein dose  (E/cm2)** |
| 0 | 0.81 | 0 | 1.47 | 0 | 0 |
| 10 | 0.81 | 0.49 | 1.47 | 0.71 | 0.16 |
| 20 | 0.81 | 0.97 | 1.47 | 1.43 | 0.32 |
| 30 | 0.81 | 1.46 | 1.47 | 2.14 | 0.48 |
| 40 | 0.81 | 1.94 | 1.47 | 2.86 | 0.64 |
| 50 | 0.81 | 2.43 | 1.47 | 3.57 | 0.79 |
| 60 | 0.81 | 2.92 | 1.47 | 4.29 | 0.95 |
| 70 | 0.81 | 3.40 | 1.47 | 5.00 | 1.11 |
| 80 | 0.81 | 3.89 | 1.47 | 5.72 | 1.27 |
| 90 | 0.81 | 4.37 | 1.47 | 6.43 | 1.43 |
| 100 | 0.81 | 4.86 | 1.47 | 7.15 | 1.59 |
| 110 | 0.81 | 5.35 | 1.47 | 7.86 | 1.75 |
| 120 | 0.81 | 5.83 | 1.47 | 8.57 | 1.91 |
| Red-LED; wavelength 615 (nm) | | | | | |
| Time (min) | **Irradiance (mW/cm2)** | **Energy density (J/cm2)** | **eV** | **Photonic fluence (p.J/cm2)** | **Einstein dose  (E/cm2)** |
| 0 | 0.79 | 0 | 2.10 | 0 | 0 |
| 10 | 0.79 | 0.47 | 2.10 | 0.99 | 0.22 |
| 20 | 0.79 | 0.95 | 2.10 | 1.99 | 0.44 |
| 30 | 0.79 | 1.42 | 2.10 | 2.99 | 0.66 |
| 40 | 0.79 | 1.90 | 2.10 | 3.99 | 0.87 |
| 50 | 0.79 | 2.37 | 2.10 | 4.99 | 1.11 |
| 60 | 0.79 | 2.84 | 2.10 | 5.98 | 1.33 |
| 70 | 0.79 | 3.32 | 2.10 | 6.98 | 1.55 |
| 80 | 0.79 | 3.79 | 2.10 | 7.98 | 1.77 |
| 90 | 0.79 | 4.27 | 2.10 | 8.97 | 1.99 |
| 100 | 0.79 | 4.74 | 2.10 | 9.97 | 2.22 |
| 110 | 0.79 | 5.21 | 2.10 | 10.97 | 2.44 |
| 120 | 0.79 | 5.67 | 2.10 | 11.97 | 2.66 |

**References**

Cuomo, C. A., S. Fanning, S. Gujja, Q. Zeng, J. R. Naglik, S. G. Filler and A. P. Mitchell (2019). "Genome Sequence for Candida albicans Clinical Oral Isolate 529L." Microbiology Resource Announcements **8**(25): e00554-00519.

Dhall, A., J. Y. Tan, M. J. Oh, S. Islam, J. Kim, A. Kim and G. Hwang (2022). "A dental implant-on-a-chip for 3D modeling of host–material–pathogen interactions and therapeutic testing platforms." Lab on a Chip **22**(24): 4905-4916.

Kim, D., T. Ito, A. Hara, Y. Li, J. Kreth and H. Koo (2022). "Antagonistic interactions by a high H2O2‐producing commensal streptococcus modulate caries development by Streptococcus mutans." Molecular Oral Microbiology **37**(6): 244-255.

Kim, H.-E., Y. Liu, A. Dhall, M. Bawazir, H. Koo and G. Hwang (2020). "Synergism of Streptococcus mutans and Candida albicans reinforces biofilm maturation and acidogenicity in saliva: an in vitro study." Frontiers in cellular and infection microbiology **10**.

Kim, H., A. Dhall, Y. Liu, M. Bawazir, H. Koo and G. Hwang (2021). "Intervening in Symbiotic Cross-Kingdom Biofilm Interactions: a Binding Mechanism-Based Nonmicrobicidal Approach." Mbio **12**(3): e00651-00621.

Kim, H. E., S. Islam, M. Park, A. Kim and G. Hwang (2020). "A Comprehensive Analysis of Near‐Contact Photobiomodulation Therapy in the Host–Bacteria Interaction Model Using 3D‐Printed Modular LED Platform." Advanced Biosystems **4**(3): 1900227.

Park, M., S. Islam, H. E. Kim, J. Korostoff, M. B. Blatz, G. Hwang and A. Kim (2020). "Human Oral Motion‐Powered Smart Dental Implant (SDI) for In Situ Ambulatory Photo‐biomodulation Therapy." Advanced Healthcare Materials **9**(16): 2000658.

Young, N. C., V. Maximiano and P. R. Arany (2022). "Thermodynamic basis for comparative photobiomodulation dosing with multiple wavelengths to direct odontoblast differentiation." J Biophotonics **15**(6): e202100398.
